# Classification of Apples (*Malus × domestica* borkh.) According to Geographical Origin, Variety and Production Method Using Liquid Chromatography Mass Spectrometry and Random Forest

**DOI:** 10.3390/foods14152655

**Published:** 2025-07-29

**Authors:** Jule Hansen, Iris Fransson, Robbin Schrieck, Christof Kunert, Stephan Seifert

**Affiliations:** 1 Institute of Food Chemistry, Hamburg School of Food Science, University of Hamburg, Grindelallee 117, 20146 Hamburg, Germany; jule.hansen@uni-hamburg.de; 2 Landeslabor Schleswig-Holstein, Max-Eyth-Str. 5, 24537 Neumünster, Germany; 3 Eurofins Food Integrity Control Services GmbH, Berliner Str. 2, 27721 Ritterhude, Germany

**Keywords:** apples, food authentication, non-targeted LC-HRMS, machine learning, random forest, feature selection, omics

## Abstract

Apples are one of the most popular fruits in Germany, valued for their regional availability and health benefits. When deciding which apple to buy, several characteristics are important to consumers, including the taxonomic variety, organic cultivation and regional production. To verify that these characteristics are correctly declared, powerful analytical methods are required. In this study, ultra-high performance liquid chromatography quadrupole time-of-flight mass spectrometry (UHPLC-Q-ToF-MS) is applied in combination with random forest to 193 apple samples for the analysis of various authentication issues. Accuracies of 93.3, 85.5, 85.6 and 90% were achieved for distinguishing between German and non-German, North and South German, organic and conventional apples and for six different taxonomic varieties. Since the classification models largely use different parts of the data, which is shown by variable selection, this method is very well suited to answer different authentication issues with one analytical approach.

## 1. Introduction

According to regulation (EU) No 1169/2011 of 25 October 2011, it should be ensured that consumers are appropriately informed about the food they consume to make choices influenced by, inter alia, health, economics, environmental, social and ethical considerations. Lately, environmental awareness, e.g., for species extinction and resource depletion, has increased and consumers are more and more interested in sustainably produced food with short transport distances [1,2], and the demand for local and organic food is rising in European countries [3]. This also applies to apples, *Malus × domestica* borkh., a fruit that is grown in many parts of the world and is the most popular fruit in Germany [4]. In addition to local and organic production, the taxonomic variety is also of interest for the consumer, as this strongly influences the taste of the apple. This interest is even more present if the consumer is allergic to apples, as the allergenicity of different varieties is different [5,6]. It is therefore important to develop reliable approaches for apple authentication that enable identification of the geographical origin, production method and taxonomic variety.

Various methods have been developed to analyze apples for these different authentication issues individually. The discrimination of apple origins using inductively coupled plasma mass spectrometry (ICP-MS), for example, achieved classification accuracies of 91%, 83% and 92% for the differentiation of European and non-European, German and non-German and North and South German samples, respectively [7]. To differentiate organic from conventional apples, Song et al. developed a low-cost and non-destructive sensor system for in-line food authentication using a smartphone and a diffraction grating sheet [8]. In addition, Barberis et al. developed a non-invasive method to differentiate the varieties of apple samples using a functionalized strip in combination with gas chromatography mass spectrometry (GC-MS) [9].

To develop a method that can analyze several of these authentication issues simultaneously, untargeted approaches that examine the metabolome are promising, as it reflects the phenotype of the sample and is influenced by external factors such as the geographical and taxonomic origin as well as the production method [10,11,12]. For this analysis, GC-MS and liquid chromatography mass spectrometry (LC-MS) as well as Proton Nuclear Magnetic Resonance (^1^H-NMR) methods are suitable. Wenck et al. applied ^1^H-NMR spectroscopy to analyze several authentication issues of apples and the discrimination of seven taxonomic varieties, German and non-German samples, as well as organically and conventionally grown apples was possible with classification accuracies of 73%, 89 and 80%, respectively [13].

Compared to ^1^H-NMR, LC-MS shows higher sensitivity [14], which is why it has already been applied for the authentication of various food [15,16,17,18,19,20,21], including apple juice [22,23]. However, due to the comparatively low robustness of LC-MS [12,14], only spectra of samples measured at the same time and on the same device are compared in these applications. When processing this data, a correspondence step is usually applied in which the detected and aligned chromatographic peaks are matched between the different spectra to generate a common peak list that is unique for each processing batch. This means that the peak lists generated in different batches cannot be used for joint data analysis, preventing the measurement and evaluation of data over a longer period of time, e.g., different harvest years. To make this possible, we have developed a data processing approach called bucketing of LC-MS spectra (BOULS) [16,24] that applies a bucketing step in which the intensities of all signals in a defined area are summed up. In this way, a standardized data structure is generated independent of the processing batch. We have shown that this approach enables the prediction and addition of data from new samples that have been processed individually to an existing RF model. In addition, generating buckets compensates for technical variances, thereby reducing the need for batch correction such as Locally Estimated Scatterplot Smoothing [25], which is difficult to implement in routine analyses. For the analysis of honey, the application of BOULS enabled long-term use of LC-MS to determine the geographical origin with a classification accuracy of 94% for 126 test samples from six different countries [24].

In order to harness the analytical data and obtain classification models for the analysis of authentication issues, machine learning approaches such as artificial neural networks, support vector machines and random forests (RF) are applied. RF is a non-parametric ensemble learning algorithm based on multiple binary decision trees. Each of these trees is trained on a subset of the samples, called bootstrap samples, and the remaining samples, called out-of-the-bag samples, can be used to generate an independent out-of-bag-error (OOB-error) [26,27]. This error is equivalent to that obtained by the use of independent validation data, when no parameter optimization is conducted, which is particularly advantageous when analyzing small data sets. This and other features, such as suitability for high-dimensional data and the ability to adjust the bootstrap sample for imbalanced training data, make RF advantageous for use in food authentication based on LC-MS data [17,18,20,21,28].

In this study, we combined untargeted ultra-high performance liquid chromatography quadrupole time-of-flight mass spectrometry (UHPLC-Q-ToF-MS) with BOULS and RF for the authentication of apple samples that were measured over a period of 8 months. In addition to analyzing whether it is possible to successfully classify apples with respect to the different authentication issues of geographical origin, variety and production method using the same approach, we also analyzed whether BOULS is generally applicable in other laboratories using different LC-MS instruments than those on which it was developed. This is particularly important because this approach promises that data can be evaluated independently of these parameters, which should be verified.

## 2. Materials and Methods

### 2.1. Sample Preparation and LC-MS Data Acquisition

In this study, 193 apple samples were analyzed once, of which 57, 124 and 12 were harvested in the years 2020, 2021 and 2022, respectively. The sample preparation and LC-MS data acquisition procedures of the routine laboratory were adopted in order to provide realistic conditions for testing the general applicability of the BOULS approach. First, the apples were washed with deionized water and juiced using a centrifugal juicer (Nutri Juicer Cold Juicer) as soon as they were completely dry, and leaves and stems were removed. The samples were stored at −20 °C until the measurement. The standard operating procedure of the laboratory was followed to create realistic conditions and, accordingly, 1 mL of the defrosted samples and 1 mL of a stock solution with caffeine (CAS-no. 58-08-2), 2,5-dihydroxybenzoic acid (CAS-no. 490-79-9) and rosmarinic acid (CAS-no. 20283-92-5) in methanol (hypergrade, Merck-no. 1.06035) were homogenized and centrifuged (20 min, 4000 g, room temperature), and the upper part was filtered (IC Millex LG 0.20 µm Low Protein Binding Hydrophilic LCR (PTFE) Merck Millipore) into HPLC vials.

A 1220 Infinity II LC system in combination with 6545 ESI-Q-TOF-MS from Agilent was used as UHPLC-Q-ToF-MS-system with an endcapped-C18 column (Waters, Acquity HSS T3, 1.8 µm, 100 x 2.1 mm). The mobile phases of the UHPLC method were water (Eluent A, H_2_O bidest.) with 0.1% formic acid (Honeywell-Fluka no. 09676) and acetonitrile (Eluent B, hypergrade, Merck-no. 1.00029) with 0.1% formic acid). The flow rate was set to 0.4 mL/minute and the start gradient was 95/5 Eluent A/Eluent B. At 3 min, 9 min, 11 min and 13 min, the mobile phase was shifted to 80/20, 55/45, 0/100 and 95/5 Eluent A/Eluent B, respectively. Stoptime was 13.5 min plus 2.5 min postrun. The injection volume was 2 µL, the column temperature was 40 °C and electrospray ionization was performed with VCap 3500 V, Nozzle 300 V and 200 °C gas temperature. The samples were measured in a randomized order.

### 2.2. Data Used for the Different Authentication Issues

Depending on the information available, different data sets were built for the different authentication issues. The data set for the differentiation between German and non-German samples comprised 193 samples, from which 117 samples originated from Schleswig-Holstein (59), Hamburg (6), Lower Saxony (14) and Baden-Württemberg (38) in Germany. The 76 non-German samples originated from Chile (15), Italy (26), New Zealand (18) and South Africa (17) and were purchased in supermarkets. The data set for the differentiation of the regional origin within Germany comprised the 117 German samples, with the 79 samples originating from Schleswig-Holstein, Hamburg and Lower Saxony categorized as North German samples and the 38 samples from Baden-Württemberg categorized as South German samples. For the differentiation according to the production method, 153 samples were used. The 113 conventionally grown apple samples originated from Chile (7), Italy (22), New Zealand (16), South Africa (15), and Germany, more precisely from the federal states Schleswig-Holstein (16), Lower Saxony (4) and Baden-Württemberg (33). The 40 organically grown samples originated from Italy (1), New Zealand (1), Schleswig-Holstein (29) and Lower Saxony (9).

The data set for the determination of the taxonomic variety comprised 80 samples. The 11 samples of the variety Braeburn originated from South Africa (3), New Zealand (4), Baden Württemberg (3) and Schleswig-Holstein (1). The 12 samples of the variety Cripps Pink originated from South Africa (5), New Zealand (2), Chile (3) and Italy (2). The 12 samples of the variety Elstar originated from Baden Württemberg (8), Schleswig-Holstein (3) and Hamburg (1). The 9 Boskoop apples originated from Schleswig-Holstein (2), Lower Saxony (2), Baden Württemberg (4) and Hamburg (1). The 28 Gala apples originated from Italy (5), New Zealand (6), South Africa (4), Chile (3), Baden Württemberg (9) and Schleswig-Holstein (1). The 8 Jonagold apples originated from Lower Saxony (1), Baden Württemberg (5) and Schleswig-Holstein (2).

### 2.3. Data Processing and Analysis

Data processing and analysis were carried out in R (version 4.4.1) [29] using the BOULS approach [24] (https://github.com/AGSeifert/BOULS, accessed on 9 January 2025, requires Linux OS). BOULS is based on the xcms workflow [30] and uses the same functions for data import and retention time alignment.

Before importing the data into R, the Agilent-specific .d files were converted to open-format mzXML files using MSConvert, which is part of the ProteoWizard software package [31] (version 3.0.21078-7da1f1136 (developer build)), and the filter peakPicking was used to convert the profile data into centroided data. The Bioconductor package mzR (version 2.26.0) was used for data import into R [31,32,33,34] and the package MSnbase (version 2.18.0) [35,36] was used to load and store the data in an object compatible with those of the xcms package (version 3.14.0) [37]. Retention time alignment was conducted using the obiwarp method [38] with a bin size of 0.1 and localAlignment set to TRUE, after peak detection using the centWave algorithm [39] with the parameters peakwidth of 15 s and ppm of 5 Da. The same sample was used as the center spectrum for each processing batch.

Subsequently, the data were processed using BOULS with a bucket size of 20 s in the retention time dimension and 2 Da in the mass dimension and the data were normalized by dividing the summarized intensities of each bucket by the sum of intensities of all buckets. This parameter combination was previously determined during optimization of data processing with BOULS.

The stats package [40,41,42] (version 4.4.1) was used for PCA and the scores plots were visualized using the ggplot2 package [43] (version 3.5.1). For classification, RF was applied using the ranger package [44] (version 0.16.0) with the parameter ntree set to 5000 and the respective default settings for mtry and min.node.size (133, which is the square root of the total number of variables and 1, respectively). To compensate for class imbalance, the parameter case.weights was chosen according to the size of the respective classes. The performance of the models was evaluated by the out-of-bag (OOB) error of the obtained random forests. The Pomona package (version 1.0.1) was used for Boruta variable selection with the value “impurity_corrected” for the importance parameter, 0.01 for the *p*-value and 100 for maxRuns [45,46].

## 3. Results and Discussion

The apple samples were analyzed using LC-MS, and Figure S1 shows representative chromatograms, which were then processed and evaluated using PCA and RF.

### 3.1. Principal Component Analysis

**Figure 1:**
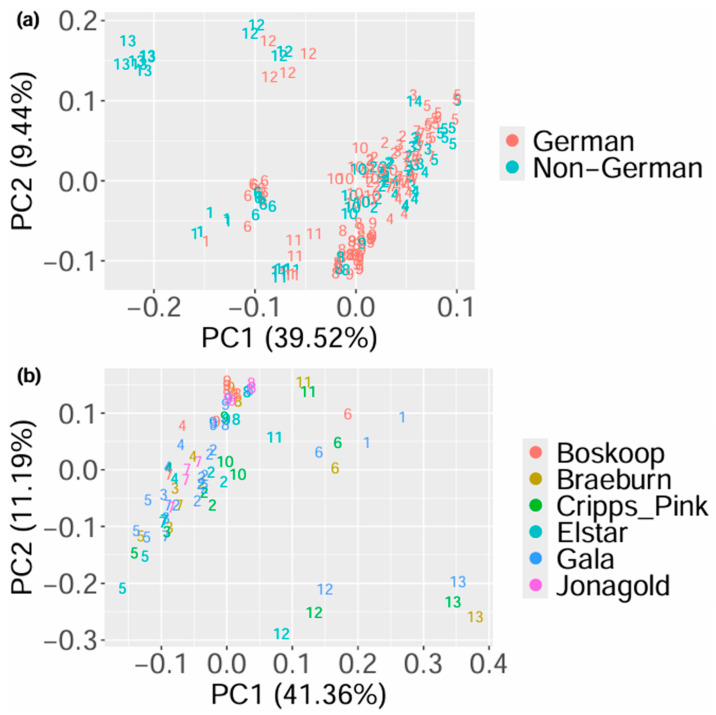
Results of the PCA showing the scores of the first and second principal component with colors according to the geographical origin (**a**) and to the taxonomic variety (**b**). The numbers correspond to the days on which the data was obtained, meaning that data points with the same number were measured on the same day.

In order to analyze the main variances of the processed data of the samples, PCA was applied, and the scores of the first two principal components labeled according to the country of origin and taxonomic variety, as well as to the region within Germany and the production method, are shown in Figure 1, Figures S2 and S3, respectively. It can be observed that the groupings are not based on the respective authentication issue but on the days on which the data was obtained. Similar results were achieved when analyzing honey using this approach [16]. They can be explained by the varying age of the columns and degree of contamination of the devices [47], which influence the data even after processing with BOULS. Similarly to the analysis of honey in the first implementation of BOULS, unsupervised data analysis cannot be applied to the analysis of apples, which is why random forest was used as a supervised method to analyze the different authentication issues.

### 3.2. Differentiation Between German and Non-German Samples

The question whether the samples originated from Germany or not arose from the assumption that it is of most importance for the consumer whether the apple originates from the country in which it is sold. Therefore, we trained a corresponding RF model and the results are shown in Table 1. In total, 8 non-German samples were misclassified as German, and 2, 5 and 1 of them originated from New Zealand, Italy and South Africa, respectively (see Table S1). The misclassification of the Italian samples could be attributed to a similarity between Italian and German apples, which has presumably arisen from an adaptation to consumer expectations, given that a large number of apples are imported from Italy to Germany every year [48]. Another possible reason for the incorrect classification is that the non-German samples were purchased in supermarkets, meaning the origin information cannot be guaranteed with certainty. However, it is highly unlikely that German samples were mislabeled, so this has negligible influence on the classification results. The overall accuracy achieved is 93.3%, which is higher than that obtained for the same authentication issue by ICP-MS (83.2%) [7] and ^1^H-NMR (88.5%) [13]. Just as in these studies, non-German samples are misclassified more frequently than German samples, which could be due to the higher within-class variance of these samples and the generally smaller number of samples. In general, the results show that LC-MS in combination with BOULS and RF is a promising approach for the identification of German apple samples.

### 3.3. Differentiation of the Regional Origin Within Germany

The regionality of food is becoming increasingly important for the consumer due to increasing environmental awareness, with a view to shorting transport distances. Therefore, it was also investigated whether North and South German samples can be distinguished, and the results are shown in Table 2. The overall accuracy is 85.5%, and 2 (of 59) and 3 (of 14) samples from Schleswig-Holstein and Lower Saxony, respectively, are misclassified (see Table S1). This could be explained by similar soil compositions for North and South German samples [49]. The reason for the much higher sensitivity of the North German class (93.7%) compared to the South German class (68.4%) could be that a smaller number of samples were used here. This may not be sufficient to adequately represent the variance of this class in the model. A similar conclusion about the influence of different sample sizes on the classification was drawn for the analysis of apple samples by ^1^H-NMR and for the analysis of honey by the approach used here [13,16,24].

The accuracy for distinguishing apples from North and South Germany is higher for LC-MS (about 85%) than for ^1^H-NMR (80.7%), but lower than for ICP-MS (92.3%). This better performance of ICP-MS could be due to soil differences, that are mainly reflected in the different element composition [50], which could also be used for the regional authentication of asparagus, almonds, walnuts, hazelnuts and truffles [51,52,53,54,55,56].

### 3.4. Differentiation Between Organically and Conventionally Produced Apples

Since the production method is also relevant for consumers, a classification model was also trained to differentiate between organically and conventionally grown apples. The results are shown in Table 3 and the model achieved an overall accuracy of 85.6%. Similarly to the distinction between apples from different regions within Germany, the sensitivity of the larger class of conventionally produced apples is much higher (97.3%) than that of the smaller class of organically produced apples, which has a sensitivity of 52.5%. As before, the difference here could be explained by the different sample sizes of the classes. However, the very high sensitivity for conventionally produced apples, which is approximately 10% higher than for the analysis of this issue with ^1^H-NMR [13], suggests that this method is very promising for application. This is because an authentication method is only needed to detect deliberate mislabeling of conventionally produced apples as organic, not vice versa, as conventional apples have lower production costs.

### 3.5. Differentiation by Taxonomic Variety

Since it is also relevant to identify the taxonomic variety of apples, especially with regard to the different allergenicity, a corresponding model was subsequently trained. The results are shown in Table 4, reaching a total accuracy of 90% for the differentiation of the six different varieties. The reason for individually occurring misclassifications could be that many of the apple varieties share common ancestry, which results in close genetic relationships [57].

Although the individual classes here are smaller than for the analyses shown above, there is no discernible influence of the corresponding class sizes on performance. The results here are therefore very different from those of the NMR analysis, where a clear influence was observed in this respect [13]. It can be concluded that the differences in the LC-MS data between the varieties are comparatively large, resulting in a fairly clear distinction.

### 3.6. Analysis of the Intersection Between the Important Variables for Different Authentication Issues

**Figure 2:**
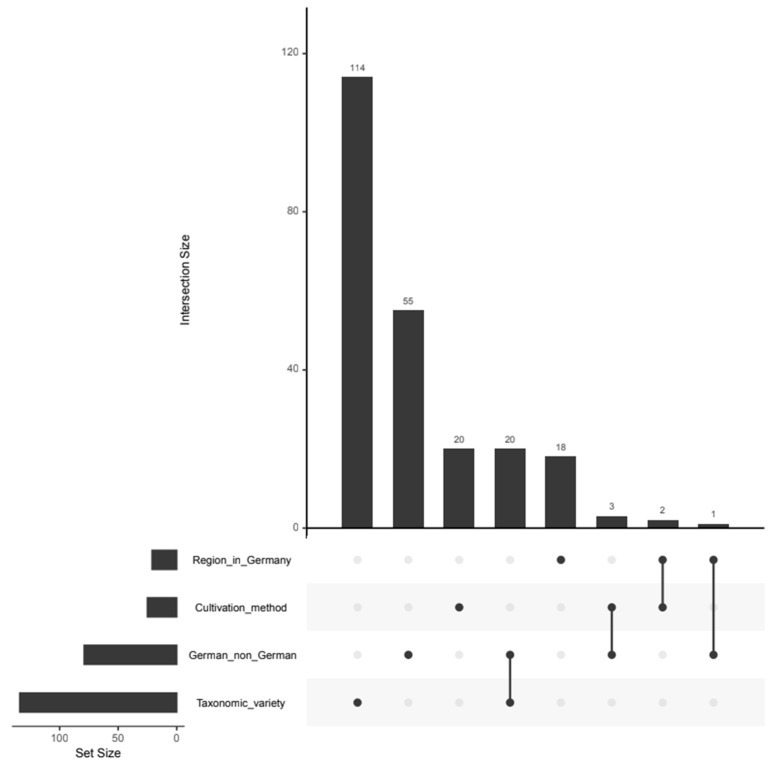
UpSet Plot showing the intersection of the selected variables for the differentiation of apples regarding the regional origin within Germany, the cultivation method, the geographical origin (German or non-German) and the taxonomic variety. The bars show the number of variables selected for a single authentication issue for single dots or multiple authentication issues for connected dots.

In order to analyze the variables on which the respective classifications are based on, variable selection was carried out. The results are shown in Tables S2–S5 and the overlap of the selected variables is shown in the UpSet plot in Figure 2. In total, 114, 55, 20 and 18 variables are individually relevant for the classification according to taxonomic variety, geographical origin, cultivation method and regional origin, respectively. The fact that most of the variables are selected for the taxonomic variety is probably due to the fact that, comparatively, many classes are distinguished there, namely six. In general, there are hardly any variables that are relevant for several classification models. However, 20 variables are selected for both the taxonomic variety and the identification of German samples. The reason for this is probably that the German and non-German samples are not evenly distributed in terms of taxonomic variety. For example, all Jonagold samples originate from Germany, while the Gala samples originate from Germany, Italy, New Zealand, South Africa and Chile (see Table S1).

## 4. Conclusions and Outlook

In this study UHPLC-Q-ToF-MS combined with BOULS data processing and RF analysis was used to differentiate apples in terms of production method, taxonomic variety, and geographical and local origin. Since high classification accuracies were achieved, especially in comparison with previously developed methods of apple authentication, and different parts of the complex data are used for the different classifications; this approach is very promising for the simultaneous analysis of different authentication issues of apples. However, as the main focus was on testing general applicability, some of the investigated groups were relatively small. In order to ensure that the full range of variation within each group is represented in the model, a larger sample size will be required for future applications. The results of this study demonstrate the general applicability of the BOULS approach to data processing, as the algorithm has been applied here in a different laboratory and to data from a different instrument manufacturer than when the approach was developed. Whether a model transfer, i.e., the application of a model developed in another laboratory, is also possible should be analyzed in a future study.

Nevertheless, this work provides the basis for the long-term application of LC-MS, e.g., in commercial or public laboratories, for the authentication of apples at different levels and other food.

## Figures and Tables

**Table 1:** Classification results of the apple data set according to the geographical origin reaching a total accuracy of 93.3% with a sensitivity of 89.5% and 95.7% for the non-German and German samples, respectively.

**True**			
**Predicted**		German	Non-German
	German	**112**	8
	Non-German	5	**68**

**Table 2:** Classification results of the apple data set according to the regionality within Germany, reaching a total accuracy of 85.5% with a sensitivity of 93.7% and 68.4% for the North and South German samples, respectively.

**True**			
**Predicted**		North	South
	North	**74**	12
	South	5	**26**

**Table 3:** Classification results of the apple data set according to the production method, reaching a total accuracy of 85.6% with a sensitivity of 97.3% and 52.5% for the conventional and biologically produced apple samples, respectively.

**True**			
**Predicted**		Conventional	Organic
	Conventional	**110**	19
	Organic	3	**21**

**Table 4:** Classification results of the apple data set according to the taxonomic variety, reaching a total accuracy of 90% and sensitivities of 88.9%, 72.7%, 100%, 83.3%, 96.4% and 87.5% for the classes Boskoop, Braeburn, Cripps Pink, Elstar, Gala and Jonagold, respectively.

**True**							
**Predicted**		Boskoop	Braeburn	Cripps Pink	Elstar	Gala	Jonagold
	Boskoop	**8**			1		
	Braeburn		**8**			3	
	Cripps Pink			**12**			
	Elstar		1		**10**	1	
	Gala		1			**27**	
	Jonagold	1					**7**

## Data Availability

The data sets analyzed during the current study are not publicly available. However, the BOULS approach is published in an R package here: https://github.com/AGSeifert/BOULS (accessed on 9 January 2025, requires Linux OS) and example data are provided here: https://www.fdr.uni-hamburg.de/record/13583 (accessed on 17 July 2025).

## Supplementary Materials

The following supporting information can be downloaded at: https://www.mdpi.com/article/10.3390/foods14152655/s1, Figure S1: Representative chromatograms of samples with the properties (a) German, Jonagold and organically produced, (b) non-German, Cripps Pink and conventionally produced, (c) non-German, Braeburn and conventionally produced, (d) non-German, Gala and conventionally produced, (e) German, Elstar and unknown production method, (f) German, Boskoop and conventionally produced; Figure S2: Results of the PCA showing the scores of the first and second principal component with colors according to the regional origin in Germany. The numbers correspond to the days on which the data was obtained; Figure S3: Results of the PCA showing the scores of the first and second principal component with colors according to the production method. The numbers correspond to the days on which the data was obtained; Table S1: Results of the RF model for the classification regarding the geographical origin of the apple samples; Table S2: Selected variables for the differentiation of German and non-German apple samples. The variable names indicate the start position of the buckets in retention time/mass level dimension; Table S3: Selected variables for the differentiation of apple samples from different origins within Germany. The variable names indicate the start position of the buckets in retention time/mass level dimension; Table S4: Selected variables for the differentiation of the different production methods. The variable names indicate the start position of the buckets in retention time/mass level dimension; Table S5: Selected variables for the differentiation according to the taxonomic variety of the apple samples. The variable names indicate the start position of the buckets in retention time/mass level dimension.
