# Supplementary material for: Classification of Apples (Malus × domestica borkh.) According to Geographical Origin, Variety and Production Method Using Liquid Chromatography Mass Spectrometry and Random Forest
Source: Foods. 2025 Jul 29;14(15):2655. doi: 10.3390/foods14152655 (PMC12345882; doi:10.3390/foods14152655)
Supplement: Supplementary file 1 [file foods-14-02655-s001.zip › Supplementary_Material.pdf]

Supplementary information

Classification of apples (*Malus* × *domestica* BORKH.) according to geographical origin, variety and production method using Liquid Chromatography Mass Spectrometry and Random Forest

Jule Hansen [1], Iris Fransson [2], Robbin Schrieck [2], Christof Kunert [3] and Stephan Seifert [1,*]

[1] Institute of Food Chemistry, Hamburg School of Food Science, University of Hamburg, Grindelallee 117, 20146 Hamburg, Germany; jule.hansen@uni-hamburg.de

[2] Landeslabor Schleswig-Holstein, Ma-Eyth-Str. 5, 24537 Neumünster, Germany; iris.fransson@lsh.landsh.de

[3] Eurofins Food Integrity Control Services GmbH, Berliner Str. 2, 27721 Ritterhude, Germany

* Correspondence: stephan.seifert@uni-hamburg.de

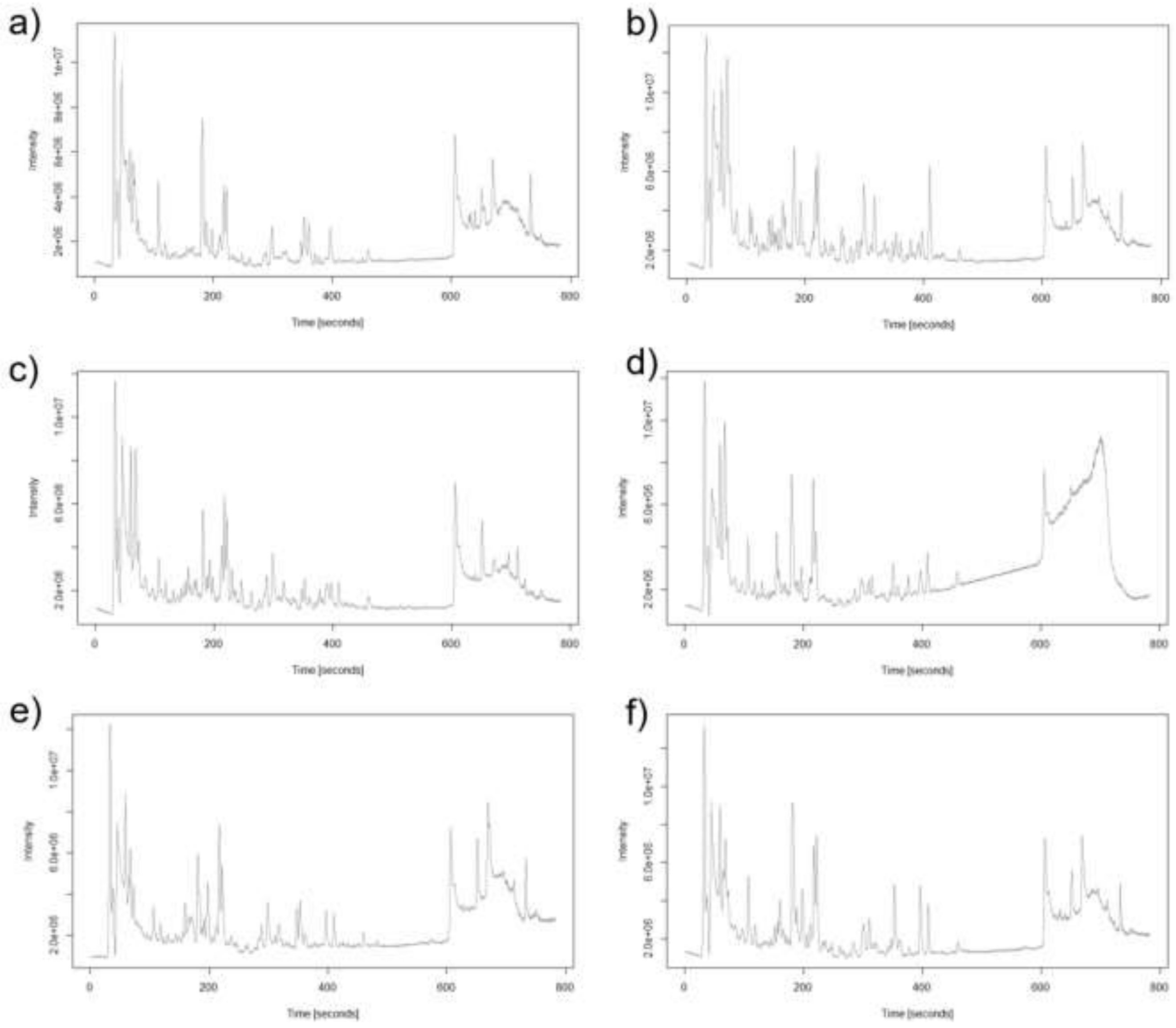

Figure S1. Representative chromatograms of samples with the properties a) German, Jonagold and organically produced, b) non-German, Cripps Pink and conventionally produced, c) non-German, Braeburn and conventionally produced, d) non-German, Gala and conventionally produced, e) German, Elstar and unknown production method, f) German, Boskoop and conventionally produced.

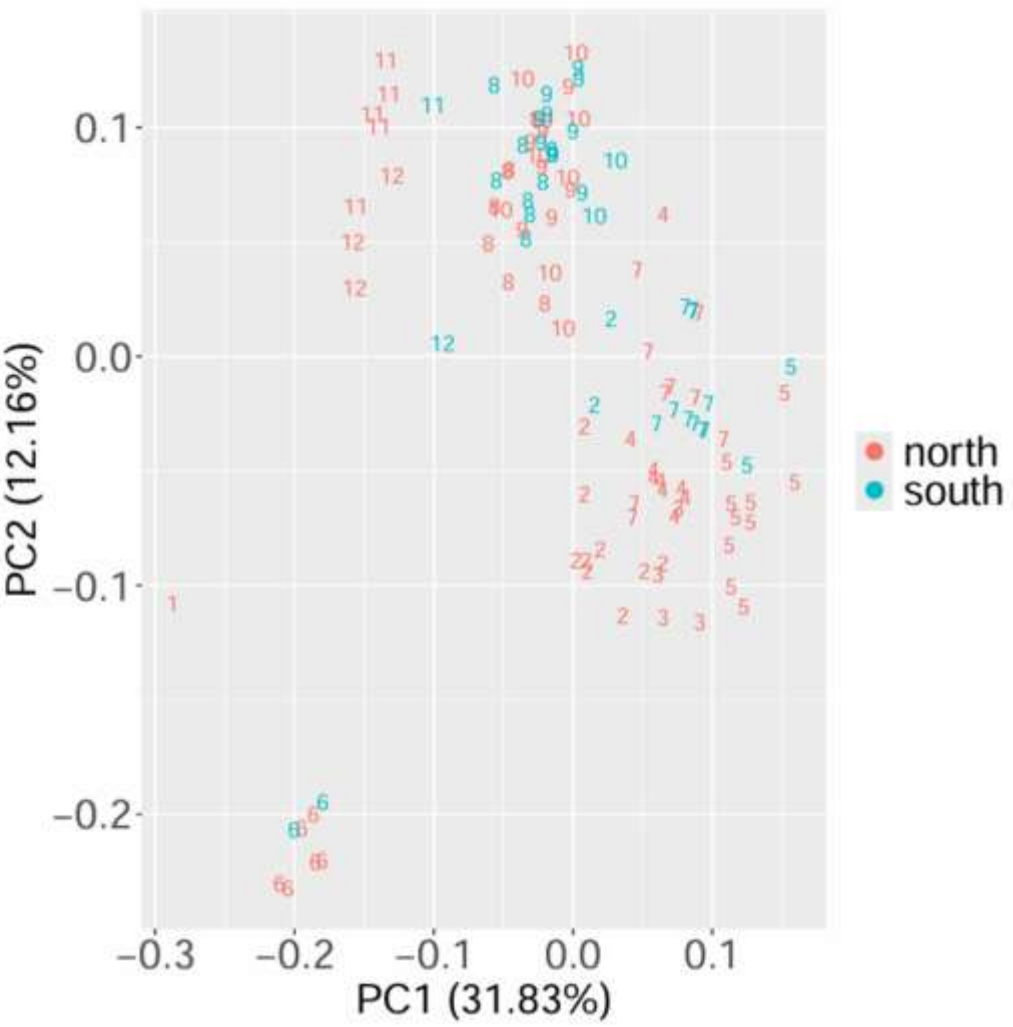

Figure S2. Results of the PCA showing the scores of the first and second principal component with colors according to the regional origin in Germany. The numbers correspond to the days on which the data was obtained.

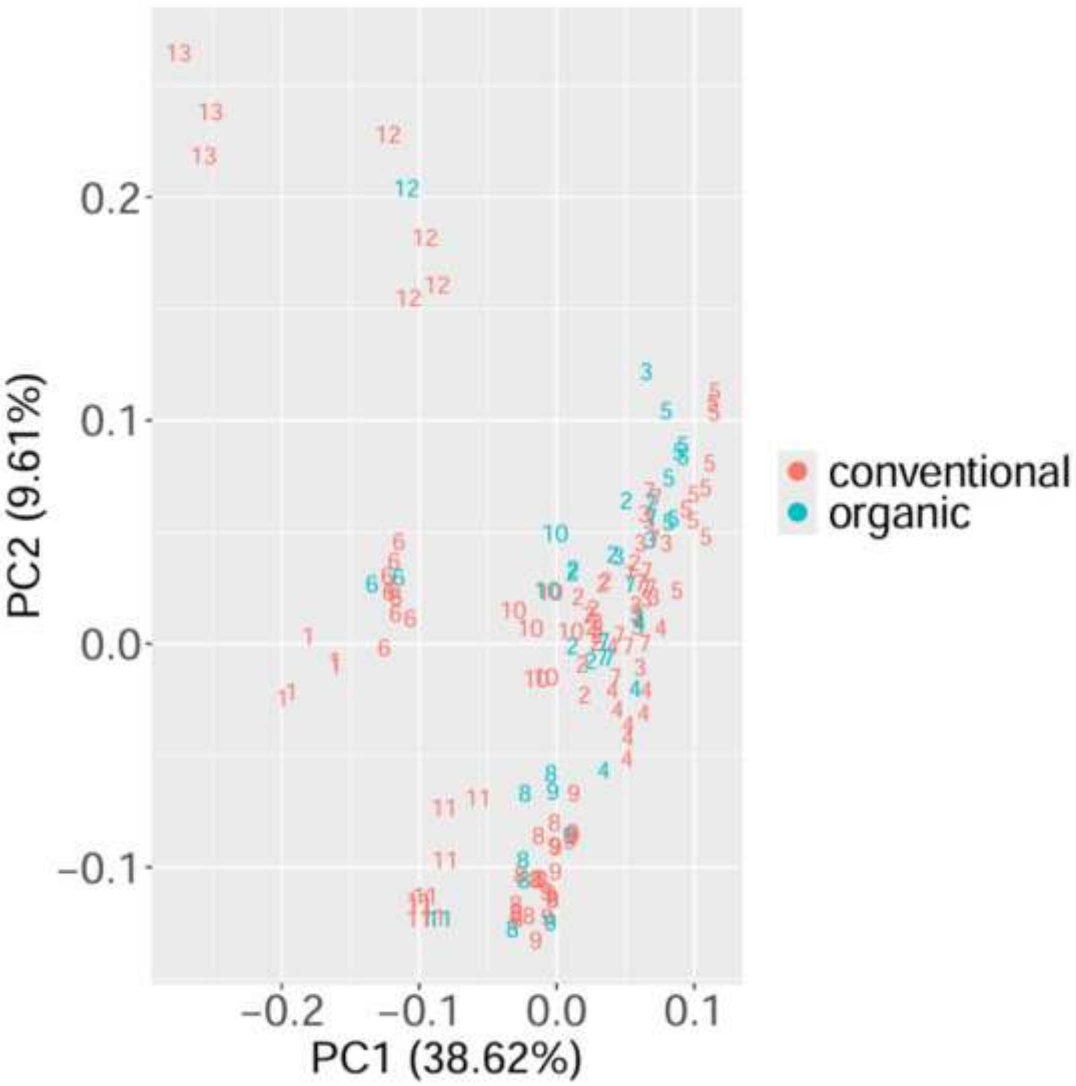

Figure S3. Results of the PCA showing the scores of the first and second principal component with colors according to the production method. The numbers correspond to the days on which the data was obtained.

Table S1. Results of the RF model for the classification regarding the geographical origin of the apple samples.

sampleID	Geogr. origin	Geographical origin		Regional origin		Production method		Taxonomic variety	
		true	predicted	true	predicted	true	pre-dicted	true	pre-dicted
SA09	South Africa	Non-German	Non-German	-	-	conventional	conventional	-	-
IT36	Italy	Non-German	Non-German	-	-	conventional	conventional	Gala	Gala
IT34	Italy	Non-German	Non-German	-	-	conventional	conventional	-	-
NZ09	New Zealand	Non-German	Non-German	-	-	conventional	conventional	Gala	Gala
SH204	Schleswig-Holstein	German	Non-German	north	north	-	-	-	-
NZ17	New Zealand	Non-German	Non-German	-	-	conventional	conventional	-	-
SH194	Schleswig-Holstein	German	German	north	north	organic	organic	-	-
SA10	South Africa	Non-German	Non-German	-	-	conventional	conventional	Gala	Gala
SA04	South Africa	Non-German	Non-German	-	-	conventional	conventional	-	-
SH232	Schleswig-Holstein	German	German	north	north	-	-	-	-
Cl04	Chile	Non-German	Non-German	-	-	-	-	-	-
SH182	Schleswig-Holstein	German	German	north	north	organic	organic	-	-
NZ10	New Zealand	Non-German	Non-German	-	-	conventional	conventional	Gala	Gala
IT27	Italy	Non-German	Non-German	-	-	conventional	conventional	Gala	Braeburn
SH183	Schleswig-Holstein	German	German	north	north	organic	organic	-	-
SH189	Schleswig-Holstein	German	German	north	north	-	-	-	-
SA07	South Africa	Non-German	Non-German	-	-	conventional	conventional	Braeburn	Braeburn
SH201	Schleswig-Holstein	German	German	north	north	-	-	-	-
NZ22	New Zealand	Non-German	Non-German	-	-	conventional	conventional	Gala	Gala

sampleID	Geogr. origin	Geographical origin		Regional origin		Production method		Taxonomic variety	
		true	predicted	true	predicted	true	pre-dicted	true	pre-dicted
BW25	Baden-Württemberg	German	Non-German	south	south	conventional	conventional	Gala	Gala
IT29	Italy	Non-German	Non-German	-	-	conventional	conventional	-	-
BW19	Baden-Württemberg	German	German	south	north	conventional	conventional	Elstar	Gala
SH203	Schleswig-Holstein	German	German	north	north	-	-	Elstar	Elstar
SA05	South Africa	Non-German	Non-German	-	-	conventional	conventional	Cripps Pink	Cripps Pink
SA11	South Africa	Non-German	Non-German	-	-	conventional	conventional	Gala	Gala
IT25	Italy	Non-German	Non-German	-	-	organic	conventional	Gala	Gala
SH213	Schleswig-Holstein	German	German	north	north	organic	organic	-	-
NZ21	New Zealand	Non-German	Non-German	-	-	conventional	conventional	-	-
SH185	Schleswig-Holstein	German	German	north	north	organic	conventional	-	-
SA12	South Africa	Non-German	Non-German	-	-	conventional	conventional	Cripps Pink	Cripps Pink
Cl02	Chile	Non-German	Non-German	-	-	-	-	Gala	Gala
SH187	Schleswig-Holstein	German	German	north	north	organic	organic	-	-
NZ08	New Zealand	Non-German	Non-German	-	-	conventional	conventional	-	-
NZ11	New Zealand	Non-German	Non-German	-	-	conventional	conventional	Braeburn	Braeburn
Cl12	Chile	Non-German	Non-German	-	-	conventional	conventional	Cripps Pink	Cripps Pink
SA08	South Africa	Non-German	Non-German	-	-	conventional	conventional	Gala	Gala
SH200	Schleswig-Holstein	German	German	north	north	-	-	-	-
IT30	Italy	Non-German	Non-German	-	-	conventional	conventional	-	-

sampleID	Geogr. origin	Geographical origin		Regional origin		Production method		Taxonomic variety	
		true	predicted	true	predicted	true	predicted	true	predicted
Cl05	Chile	Non-German	Non-German			conventional	conventional		
SH184	Schleswig-Holstein	German	German	north	north	organic	organic	-	-
IT22	Italy	Non-German	Non-German	-	-	conventional	conventional	-	-
SA06	South Africa	Non-German	Non-German	-	-	conventional	conventional	Braeburn	Gala
NZ16	New Zealand	Non-German	Non-German	-	-	organic	conventional	Gala	Gala
SH198	Schleswig-Holstein	German	German	north	north	organic	organic	-	-
IT24	Italy	Non-German	Non-German	-	-	conventional	conventional	-	-
Cl07	Chile	Non-German	Non-German	-	-	conventional	conventional	-	-
NZ07	New Zealand	Non-German	German	-	-	conventional	conventional	Braeburn	Braeburn
SH205	Schleswig-Holstein	German	German	north	north	organic	organic	-	-
NZ15	New Zealand	Non-German	Non-German	-	-	conventional	conventional	-	-
HH18	Hamburg	German	German	north	north	-	-	Elstar	Elstar
HH19	Hamburg	German	German	north	north	-	-	-	-
SH191	Schleswig-Holstein	German	German	north	north	-	-	-	-
IT26	Italy	Non-German	Non-German	-	-	conventional	conventional	Cripps Pink	Cripps Pink
SH227	Schleswig-Holstein	German	German	north	north	conventional	organic	Boskoop	Boskoop
NZ19	New Zealand	Non-German	Non-German	-	-	conventional	conventional	Gala	Gala
SH212	Schleswig-Holstein	German	German	north	north	organic	conventional	-	-
IT32	Italy	Non-German	Non-German	-	-	convnetional	conventional	-	-
NZ18	New Zealand	Non-German	Non-German	-	-	convnetional	conventional	Gala	Gala

sampleID	Geogr. origin	Geographical origin		Regional origin		Production method		Taxonomic variety	
		true	predicted	true	predicted	true	pre-dicted	true	pre-dicted
SH230	Schles-wig-Holstein	German	German	north	north	conven-tional	organic	-	-
IT33	Italy	Non-Ger-man	Non-Ger-man	-	-	convne-tional	conven-tional	-	-
SH209	Schles-wig-Holstein	German	German	north	south	organic	conven-tional	-	-
SH188	Schles-wig-Holstein	German	German	north	north	-	-	-	-
SH186	Schles-wig-Holstein	German	German	north	north	organic	organic	-	-
IT28	Italy	Non-Ger-man	Non-Ger-man	-	-	conven-tional	conven-tional	-	-
IT31	Italy	Non-Ger-man	Non-Ger-man	-	-	conven-tional	conven-tional	-	-
SH229	Schles-wig-Holstein	German	German	north	north	conven-tional	organic		
SH231	Schles-wig-Holstein	German	German	north	north	organic	organic	-	-
BW22	Baden-Würt-temberg	German	German	south	north	-	-	Gala	Gala
BW23	Baden-Würt-temberg	German	German	south	north	conven-tional	conven-tional	Elstar	Elstar
NI37	Lower Saony	German	German	north	south	conven-tional	conven-tional	-	-
IT38	Italy	Non-Ger-man	Non-Ger-man	-	-	conven-tional	conven-tional	Gala	Gala
Cl06	Chile	Non-Ger-man	Non-Ger-man	-	-	conven-tional	conven-tional	Cripps Pink	Cripps Pink
SH206	Schles-wig-Holstein	German	German	north	north	organic	conven-tional	-	-
SH207	Schles-wig-Holstein	German	German	north	north	organic	organic	Elstar	Elstar
SH199	Schles-wig-Holstein	German	German	north	north	organic	organic	-	-

sampleID	Geogr. origin	Geographical origin		Regional origin		Production method		Taxonomic variety	
		true	predicted	true	predicted	true	pre-dicted	true	pre-dicted
Cl08	Chile	Non-German	Non-German	-	-	conventional	conventional	Gala	Gala
SH193	Schleswig-Holstein	German	German	north	north	organic	organic	-	-
IT35	Italy	Non-German	Non-German	-	-	conventional	conventional	-	-
SH192	Schleswig-Holstein	German	German	north	north	organic	organic	-	-
NZ13	New Zealand	Non-German	Non-German	-	-	conventional	conventional	Braeburn	Gala
HH17	Hamburg	German	German	north	north	-	-	-	-
SH197	Schleswig-Holstein	German	German	north	north	organic	conventional	-	-
SH202	Schleswig-Holstein	German	German	north	north	-	-	-	-
Cl10	Chile	Non-German	Non-German	-	-	conventional	conventional	Cripps Pink	Cripps Pink
SH208	Schleswig-Holstein	German	German	north	north	organic	conventional	-	-
BW20	Baden-Württemberg	German	German	south	north	conventional	conventional	-	-
NZ12	New Zealand	Non-German	Non-German	-	-	conventional	conventional	-	-
NI38	Lower Saony	German	German	north	north	conventional	conventional	-	-
SH228	Schleswig-Holstein	German	German	north	north	conventional	conventional	-	-
NZ20	New Zealand	Non-German	Non-German	-	-	conventional	conventional	-	-
HH16	Hamburg	German	German	north	north	-	-	Boskoop	Boskoop
IT23	Italy	Non-German	Non-German	-	-	conventional	conventional	-	-
BW21	Baden-Württemberg	German	German	south	north	-	-	-	-
NZ14	New Zealand	Non-German	Non-German	-	-	conventional	conventional	Braeburn	Braeburn

sampleID	Geogr. origin	Geographical origin		Regional origin		Production method		Taxonomic variety	
		true	predicted	true	predicted	true	predicted	true	predicted
Cl09	Chile	Non-German	Non-German	-	-	conventional	conventional	Gala	Gala
BW40	Baden-Württemberg	German	German	south	south	conventional	conventional	Gala	Gala
BW34	Baden-Württemberg	German	German	south	south	conventional	conventional	-	-
NI31	Lower Saony	German	German	north	north	organic	organic	-	-
BW47	Baden-Württemberg	German	German	south	south	conventional	conventional	Galar	Elstar
BW29	Baden-Württemberg	German	German	south	north	conventional	conventional	Boskoop	Boskoop
HH15	Hamburg	German	German	north	north	-	-	-	-
SH114	Schleswig-Holstein	German	German	north	north	conventional	conventional	Jonagold	Jonagold
BW33	Baden-Württemberg	German	German	south	south	conventional	conventional	Jonagold	Jonagold
NI26	Lower Saony	German	German	north	north	organic	conventional	-	-
BW35	Baden-Württemberg	German	German	south	north	conventional	conventional	Braeburn	Braeburn
IT20	Italy	Non-German	German	-	-	-	-	-	-
NI2	Lower Saony	German	German	north	north	organic	organic	Jonagold	Boskoop
NI32	Lower Saony	German	German	north	north	organic	conventional	-	-
BW52	Baden-Württemberg	German	German	south	north	conventional	conventional	Jonagold	Jonagold
SH164	Schleswig-Holstein	German	German	north	north	organic	organic	-	-
SH159	Schleswig-Holstein	German	German	north	north	organic	organic	-	-

sampleID	Geogr. origin	Geographical origin		Regional origin		Production method		Taxonomic variety	
		true	predicted	true	predicted	true	pre-dicted	true	pre-dicted
BW54	Baden-Würt-temberg	German	German	south	south	conven-tional	conven-tional	-	-
SH122	Schles-wig-Holstein	German	German	north	north	conven-tional	conven-tional	-	-
BW51	Baden-Würt-temberg	German	German	south	south	conven-tional	conven-tional	Gala	Gala
BW37	Baden-Würt-temberg	German	German	south	south	conven-tional	conven-tional	Elstar	Elstar
BW43	Baden-Würt-temberg	German	German	south	south	conven-tional	conven-tional	Brae-burn	Brae-burn
BW32	Baden-Würt-temberg	German	German	south	south	conven-tional	conven-tional	Bos-koop	Bos-koop
SH72	Schles-wig-Holstein	German	German	north	north	organic	conven-tional	-	-
BW36	Baden-Würt-temberg	German	German	south	south	conven-tional	conven-tional	Gala	Gala
IT10	Italy	Non-Ger-man	Non-Ger-man	-	-	conven-tional	conven-tional	-	-
SH171	Schles-wig-Holstein	German	German	north	north	organic	conven-tional	Elstar	Brae-burn
IT11	Italy	Non-Ger-man	Non-Ger-man	-	-	conven-tional	conven-tional	-	-
SH128	Schles-wig-Holstein	German	German	north	north	-	-	-	-
SH7	Schles-wig-Holstein	German	German	north	north	organic	organic	-	-
SH178	Schles-wig-Holstein	German	German	north	north	organic	conven-tional	-	-
BW45	Baden-Würt-temberg	German	German	south	south	conven-tional	conven-tional	Bos-koop	Bos-koop
BW53	Baden-Würt-temberg	German	German	south	south	conven-tional	conven-tional	Elstar	Elstar

sampleID	Geogr. origin	Geographical origin		Regional origin		Production method		Taxonomic variety	
		true	predicted	true	predicted	true	predicted	true	predicted
BW48	Baden-Württemberg	German	German	south	south	conventional	conventional	Jonagold	Jonagold
SH134	Schleswig-Holstein	German	German	north	north	conventional	conventional	Jonagold	Jonagold
NI28	Lower Saony	German	German	north	north	organic	conventional	-	-
BW39	Baden-Württemberg	German	German	south	south	conventional	conventional	Boskoop	Boskoop
IT1	Italy	Non-German	German	-	-	conventional	conventional	Gala	Gala
BW28	Baden-Württemberg	German	German	south	south	conventional	conventional	-	-
BW30	Baden-Württemberg	German	German	south	south	conventional	conventional	Braeburn	Braeburn
BW31	Baden-Württemberg	German	German	south	south	conventional	conventional	Gala	Gala
SH102	Schleswig-Holstein	German	German	north	south	-	-	-	-
BW50	Baden-Württemberg	German	German	south	south	conventional	conventional	Elstar	Elstar
SA1	South Africa	Non-German	German	-	-	conventional	conventional	Cripps Pink	Cripps Pink
SH105	Schleswig-Holstein	German	German	north	north	-	-	Boskoop	Boskoop
BW38	Baden-Württemberg	German	German	south	south	conventional	conventional	Jonagold	Jonagold
NI30	Lower Saony	German	German	north	north	conventional	conventional	-	-
NI21	Lower Saony	German	German	north	south	-	-	-	-
BW46	Baden-Württemberg	German	German	south	south	conventional	conventional	Gala	Gala
BW44	Baden-Württemberg	German	German	south	south	conventional	conventional	-	-

sampleID	Geogr. origin	Geographical origin		Regional origin		Production method		Taxonomic variety	
		true	predicted	true	predicted	true	pre-dicted	true	pre-dicted
BW41	Baden-Würt-temberg	German	German	south	south	conven-tional	conven-tional	Gala	Gala
NI7	Lower Saony	German	German	north	south	organic	conven-tional	Bos-koop	Elstar
BW42	Baden-Würt-temberg	German	German	south	south	conven-tional	conven-tional	Jonag-old	Jonag-old
BW49	Baden-Würt-temberg	German	German	south	south	conven-tional	conven-tional	Gala	Gala
SH161	Schles-wig-Holstein	German	German	north	north	organic	organic	-	-
NI9	Lower Saony	German	German	north	north	organic	conven-tional	Bos-koop	Bos-koop
BW27	Baden-Würt-temberg	German	German	south	south	conven-tional	conven-tional	-	-
BW5	Baden-Würt-temberg	German	German	south	north	-	-	-	-
HH14	Ham-burg	German	German	north	north	-	-	-	-
SH129	Schles-wig-Holstein	German	German	north	north	-	-	-	-
SH127	Schles-wig-Holstein	German	German	north	north	-	-	-	-
NZ5	New Zealand	Non-Ger-man	German	-	-	-	-	Cripps Pink	Cripps Pink
CL1	Chile	Non-Ger-man	Non-Ger-man	-	-	-	-	-	-
SH144	Schles-wig-Holstein	German	German	north	north	conven-tional	conven-tional	-	-
NI34	Lower Saony	German	German	north	north	conven-tional	conven-tional	-	-
IT21	Italy	Non-Ger-man	Non-Ger-man	-	-	-	-	-	-
BW2	Baden-Würt-temberg	German	German	south	north	-	-	-	-
NZ2	New Zealand	Non-Ger-man	Non-Ger-man	-	-	conven-tional	conven-tional	Cripps Pink	Cripps Pink

sampleID	Geogr. origin	Geographical origin		Regional origin		Production method		Taxonomic variety	
		true	predicted	true	predicted	true	pre-dicted	true	pre-dicted
SH71	Schles-wig-Holstein	German	German	north	north	organic	organic	-	-
SH138	Schles-wig-Holstein	German	German	north	north	conven-tional	conven-tional	-	-
SH74	Schles-wig-Holstein	German	German	north	north	organic	conven-tional	-	-
IT2	Italy	Non-Ger-man	German	-	-	conven-tional	conven-tional	-	-
SH140	Schles-wig-Holstein	German	German	north	north	conven-tional	conven-tional	-	-
SH111	Schles-wig-Holstein	German	Non-Ger-man	north	north	conven-tional	conven-tional	Brae-burn	Brae-burn
SH37	Schles-wig-Holstein	German	German	north	north	conven-tional	conven-tional	-	-
IT5	Italy	Non-Ger-man	German	-	-	conven-tional	conven-tional	-	-
IT15	Italy	Non-Ger-man	Non-Ger-man	-	-	conven-tional	conven-tional	-	-
IT19	Italy	Non-Ger-man	German	-	-	-	-	-	-
IT18	Italy	Non-Ger-man	Non-Ger-man	-	-	conven-tional	conven-tional	Cripps Pink	Cripps Pink
SH142	Schles-wig-Holstein	German	German	north	north	conven-tional	conven-tional	-	-
BW24	Baden-Würt-temberg	German	German	south	north	conven-tional	conven-tional	Elstar	Elstar
SH117	Schles-wig-Holstein	German	German	north	north	conven-tional	conven-tional	-	-
NI13	Lower Saony	German	German	north	north	organic	conven-tional	-	-
BW7	Baden-Würt-temberg	German	German	south	north	-	-	Elstar	Elstar
SH145	Schles-wig-Holstein	German	Non-Ger-man	north	north	conven-tional	conven-tional	-	-

sampleID	Geogr. origin	Geographical origin		Regional origin		Production method		Taxonomic variety	
		true	predicted	true	predicted	true	pre-dicted	true	pre-dicted
SA13	South Africa	Non-German	Non-German	-	-	conventional	conventional	Cripps Pink	Cripps Pink
SH109	Schleswig-Holstein	German	Non-German	north	north	conventional	conventional	Gala	Gala
CL18	Chile	Non-German	Non-German	-	-	-	-	-	-
NI12	Lower Saony	German	German	north	north	organic	conventional	-	-
SA016	South Africa	Non-German	Non-German	-	-	-	-	-	-
SA19	South Africa	Non-German	Non-German	-	-	conventional	conventional	-	-
SA23	South Africa	Non-German	Non-German	-	-	conventional	conventional	Gala	Gala
CL20	Chile	Non-German	Non-German	-	-	-	-	-	-
CL22	Chile	Non-German	Non-German	-	-	-	-	-	-
SA22	South Africa	Non-German	Non-German	-	-	conventional	conventional	Braeburn	Gala
CL15	Chile	Non-German	Non-German	-	-	-	-	-	-
SA24	South Africa	Non-German	Non-German	-	-	conventional	conventional	Cripps Pink	Cripps Pink
CL17	Chile	Non-German	Non-German	-	-	-	-	-	-
SA015	South Africa	Non-German	Non-German	-	-	-	-	-	-

Table S2. Selected variables for the differentiation of German and non-German apple samples. The variable names indicate the start position of the buckets in retention time / mass level dimension.

Selected variables
580 / 58
300 / 66
300 / 82
60 / 92
60 / 96
300 / 108
300 / 126
340 / 128
60 / 136
340 / 146
200 / 152
340 / 152
220 / 158
180 / 176
220 / 176
140 / 188
340 / 188
480 / 190
140 / 204
140 / 206
480 / 208
360 / 222
100 / 228
620 / 230
200 / 242
440 / 246
360 / 250
80 / 254
440 / 264
100 / 266
180 / 268
340 / 274
340 / 276
100 / 284
100 / 288
100 / 290
60 / 294
160 / 306
340 / 308
60 / 312
520 / 314
600 / 342
60 / 344
220 / 352

Selected variables
100 / 366
100 / 368
180 / 370
220 / 370
160 / 384
200 / 400
340 / 410
100 / 420
160 / 420
340 / 428
360 / 430
340 / 438
480 / 442
100 / 446
480 / 446
340 / 486
340 / 488
100 / 498
60 / 502
240 / 502
140 / 508
280 / 564
340 / 576
400 / 592
400 / 594
300 / 608
220 / 762
340 / 842
220 / 860
340 / 874
340 / 898
120 / 922
340 / 922
400 / 964

Table S3. Selected variables for the differentiation of apple samples from different origins within Germany. The variable names indicate the start position of the buckets in retention time / mass level dimension.

Selected variables
80 / 60
80 / 174
620 / 230
260 / 238
60 / 240
60 / 246
60 / 256
360 / 268
60 / 270
500 / 278
460 / 304
60 / 348
360 / 452
120 / 616
80 / 618
80 / 622
80 / 638
320 / 652
80 / 654
120 / 660
180 / 660
360 / 860
720 / 976
720 / 992
660 / 998
680 / 998

Table S4. Selected variables for the differentiation of the different production methods. The variable names indicate the start position of the buckets in retention time / mass level dimension.

**Selected variables**
440 / 64
120 / 170
120 / 188
480 / 190
480 / 208
360 / 250
360 / 252
60 / 268
80 / 268
360 / 268
60 / 270
360 / 270
180 / 272
140 / 364
100 / 374
200 / 380
120 / 418
80 / 514
80 / 530
80 / 544
220 / 698
80 / 708
200 / 736
400 / 950
340 / 966
380 / 966

Table S5. Selected variables for the differentiation according to the taxonomic variety of the apple samples. The variable names indicate the start position of the buckets in retention time / mass level dimension.

Selected variables
380 / 164
200 / 176
220 / 176
380 / 180
100 / 188
340 / 188
120 / 196
180 / 196
120 / 212
60 / 220
160 / 254
380 / 260
60 / 278
360 / 302
360 / 304
100 / 306
180 / 306
340 / 308
140 / 320
160 / 320
160 / 322
200 / 330
80 / 332
340 / 332
220 / 334
220 / 336
380 / 344
380 / 346
320 / 348
340 / 348
380 / 348
380 / 350
220 / 352
80 / 354
160 / 356
220 / 356
180 / 358
100 / 366
360 / 366
380 / 366
100 / 368
220 / 370
80 / 374
220 / 376

Selected variables
120 / 378
300 / 380
120 / 382
300 / 384
320 / 384
340 / 384
280 / 386
300 / 386
380 / 388
80 / 390
120 / 396
180 / 398
380 / 398
200 / 400
200 / 404
260 / 404
360 / 404
80 / 408
380 / 408
340 / 410
80 / 412
340 / 412
180 / 414
100 / 420
120 / 420
160 / 420
340 / 428
340 / 432
220 / 434
240 / 434
180 / 438
380 / 442
480 / 442
380 / 444
320 / 446
480 / 446
340 / 448
360 / 450
340 / 476
360 / 476
340 / 478
360 / 478
340 / 480
360 / 480
360 / 482
340 / 486

Selected variables
340 / 488
360 / 490
340 / 496
360 / 510
340 / 512
360 / 512
380 / 516
380 / 518
500 / 526
320 / 550
320 / 552
280 / 576
420 / 590
140 / 596
460 / 606
320 / 608
320 / 610
160 / 624
80 / 642
380 / 642
340 / 644
380 / 646
380 / 672
140 / 674
380 / 674
140 / 676
380 / 676
160 / 698
220 / 726
220 / 728
200 / 734
340 / 838
340 / 842
340 / 844
200 / 850
340 / 874
60 / 888
340 / 898
60 / 934
